# Upgrading SELEX Technology by Using Lambda Exonuclease Digestion for Single-Stranded DNA Generation

**DOI:** 10.3390/molecules15010001

**Published:** 2009-12-24

**Authors:** Meltem Avci-Adali, Angela Paul, Nadja Wilhelm, Gerhard Ziemer, Hans Peter Wendel

**Affiliations:** Department of Thoracic, Cardiac, and Vascular Surgery, University Hospital Tuebingen, Calwerstr. 7/1, Tuebingen 72076, Germany

**Keywords:** aptamers, strand separation, streptavidin-coated beads, lambda exonuclease, single-stranded DNA

## Abstract

The generation of single-stranded DNA (ssDNA) molecules plays a key role in the SELEX (Systematic Evolution of Ligands by EXponential enrichment) combinatorial chemistry process and numerous molecular biology techniques and applications, such as DNA sequencing, single-nucleotide polymorphism (SNP) analysis, DNA chips, DNA single-strand conformation polymorphism (SSCP) analysis and many other techniques. The purity and yield of ssDNA can affect the success of each application. This study compares the two ssDNA production methods, the strand separation by streptavidin-coated magnetic beads and alkaline denaturation and the lambda exonuclease digestion, in regard to the purity of generated ssDNA and the efficiency. Here, we demonstrate the considerable benefits of ssDNA production by lambda exonuclease digestion for *in vitro* selection of DNA aptamers. We believe that the generation of ssDNA aptamers using this method will greatly improve the success rate of SELEX experiments concerning the recovery of target-specific aptamers.

## Introduction

Single-stranded DNA (ssDNA) templates are required during the SELEX (Systematic Evolution of Ligands by EXponential enrichment) combinatorial chemistry process for ssDNA aptamer generation [1,2,3,4] and for many other molecular biology and biotechnology applications including sensors, DNA chips [5] and microarrays, pyrosequencing technology [6], detection of point mutations in DNA using the single-stranded conformation polymorphism technique (SSCP) [7], single-nucleotide polymorphism (SNP) analysis [8], solid phase DNA sequencing [9,10], and multitude of other molecular techniques. Several methods exist for the generation of ssDNA from double stranded DNA (dsDNA), including denaturing urea-polyacrylamide gel [11,12,13,14], asymmetric PCR [15,16], lambda exonuclease digestion [17,18] and magnetic separation with streptavidin-coated beads [9,19]. For strand separation with denaturing PAGE, PCR is performed with a primer that contains a spacer as terminator (e.g., hexaethylene glycol (HEGL)) and an extension of 20 adenine nucleotides (polyA) to produce strands with different lengths. After PCR, the dsDNA with unequal strands is separated by denaturing gel electrophoresis. The desired ssDNA strand has to be eluted from the polyacrylamide gel. Asymmetric PCR is used to preferentially amplify one strand of the original DNA more than the other. Therefore, PCR is performed with unequal molar ratio of forward and reverse primers. As asymmetric PCR proceeds, the lower concentration primer is incorporated into dsDNA and the higher concentration primer is used to synthesize an excess of ssDNA in each cycle. However, asymmetric PCR products comprise not only single but also double stranded DNA. Thus, the PCR products have to be separated by non-denaturing PAGE and the ssDNA has to be eluted from the gel. The purification of small ssDNA fragments from polyacrylamide gels is very time-consuming and results in poor yield of ssDNA which is during SELEX experiments undesirable due to massive loss of target binding aptamers. Therefore, the strand separation by denaturing PAGE and asymmetric PCR with subsequent purification of ssDNA from non-denaturing polyacrylamide gels are not recommendable for SELEX experiments, especially in the first selection rounds in which the binding sequences are not sufficiently amplified. The most widely used method for ssDNA generation is the immobilization of biotinylated dsDNA onto streptavidin-coated beads and the denaturation of dsDNA by alkaline treatment. During PCR, one of the two primers is biotinylated. Afterwards, the biotinylated PCR product is immobilized onto streptavidin-coated beads. The desired non-biotinylated ssDNA is separated from biotinylated strand by alkaline denaturation. Thereby, biotinylated strand should remain bead-bound via binding to streptavidin. However, our previous studies demonstrated the dissociation of streptavidin by alkaline treatment [20]. Thereby, undesired biotinylated strands enter into the eluate and can re-anneal to the complementary strands and lose the tertiary structure which is important for the target binding ability. Furthermore, the streptavidin in the aptamer pool presents an additional target for aptamers during SELEX process. Hitherto, several groups successfully generated aptamers against streptavidin [13,21,22]. An alternative method to strand separation with streptavidin-coated magnetic beads is the lambda exonuclease digestion of undesired strand. Lambda exonuclease is a highly processive 5´→3´ exodeoxyribonuclease that selectively digests the 5´-phosphorylated strand of dsDNA. For this purpose, a 5´-phosphate group is introduced into one strand of dsDNA by performing PCR where only one of the two primers is 5´-phoshorylated. The phosphorylated strand is then removed by digestion with lambda exonuclease.

The preparation of high-quality ssDNA is not only important for the success of SELEX technology but also for other applications. Therefore, we investigated the purity of generated ssDNA after the most commonly used method “streptavidin-coated magnetic beads and alkaline treatment” and the auspicious alternative method “lambda exonuclease digestion”. Furthermore, the efficiency of both ssDNA generation methods was compared.

## Results and Discussion

### Detection of biotinylated DNA strand after strand separation with streptavidin-coated magnetic beads and alkaline denaturation

**Figure 1:**
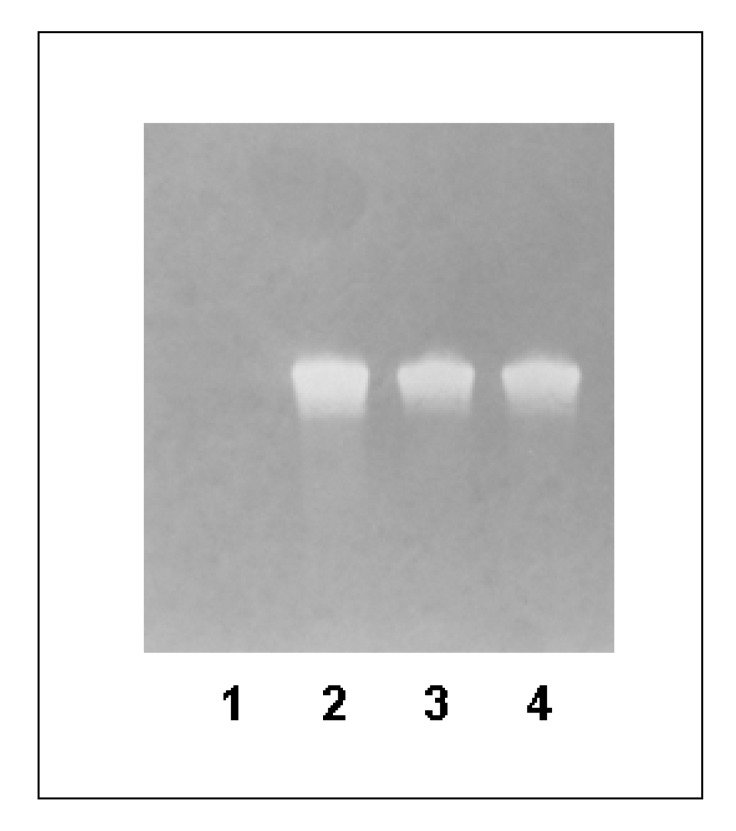
Detection of biotinylated DNA strand by modified Western blot.

The biotinylated dsDNA was immobilized onto the streptavidin-coated magnetic beads. Subsequently, the beads with dsDNA were incubated with 0.15 M NaOH to separate the DNA strands. The ssDNA amount after five independent strand separation experiments with streptavidin-coated magnetic beads was determined. The average yield was 21.1 ± 9.1% from the maximum possible ssDNA amount. The obtained ssDNA samples were separated by denaturing PAGE (10% TBE-Urea gel) and transferred onto positively charged nylon membrane. Biotinylated ssDNA was detected by incubation with an alkaline-phosphatase conjugated anti-biotin antibody. Using this method, we demonstrated that after strand separation the eluate is contaminated with undesired biotinylated DNA strand (Figure 1). A biotinylated ssDNA aptamer was used as positive control (Figure 1, lane 4). As expected, the ssDNA library (SB) without biotin labeling showed no anti-biotin antibody binding (Figure 1, lane 1). The biotinylated dsDNA without strand separation (Figure 1, lane 2) as well as the eluate after strand separation were positive for biotinylated complementary strand (Figure 1, lane 3).

Samples were separated on a 10% TBE-Urea gel and transferred onto a positively charged nylon membrane. Biotinylated ssDNA was detected using alkaline phosphatase-conjugated anti-biotin antibody. Lane (1) 1.3 µg unlabeled ssDNA library (SB), (2) 1.3 µg dsDNA prior to strand separation, (3) 1.3 µg ssDNA after strand separation, (4) 1.3 µg biotinylated ssDNA aptamer.

The strand separation with streptavidin-coated magnetic beads and alkaline denaturation resulted in an ssDNA product which was contaminated with the non-desired complementary (biotinylated) strand as well as with streptavidin as proven in our previous studies [20]. The presence of complementary strand can lead to the re-hybridization of both strands and thereby to the loss of binding ability of selected aptamers to the target. Thus, specific aptamer sequences that were able to bind target before will be lost. Furthermore, the streptavidin in the aptamer pool presents an additional SELEX target and triggers the aggregation of cells, most likely via its RGD mimicking sequence [23], if performing cell-SELEX [20]. The clustering of cells by streptavidin disables the partitioning of non-binding aptamer sequences form binding sequences and impedes the selection process.

### Detection of phosphorylated DNA strand after lambda exonuclease digestion

**Figure 2:**
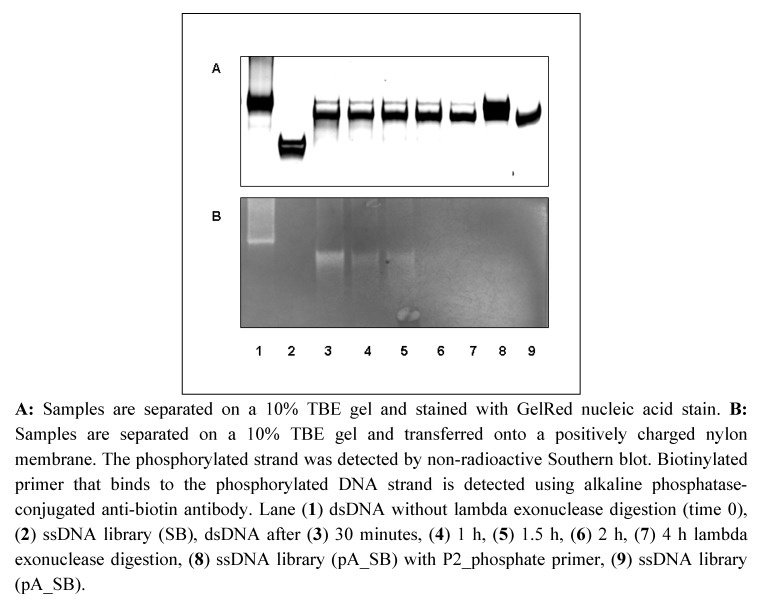
Digestion of the phosphorylated strand of dsDNA with 25 U lambda exonuclease and different digestion times.

PCR was performed with a 5´-phosphate labeled reverse primer (P2_phosphate) and polyA (20) labeled forward primer (P1_pA). The undesired complementary DNA strand with phosphate labeling is digested by lambda exonuclease. The digestion was performed with 6.6 µg DNA per reaction sample and stopped after different incubation times (0, 30, 60, 90, 120, and 240 minutes) at 37 °C. The non-denaturing PAGE (Figure 2A) demonstrated that the reaction mixtures contain mainly polyA strands (Figure 2A, lane 3-7). But a weak band was also visible at the same height as the double-stranded DNA (Figure 2A, lane 1). Although initially an incomplete digestion has been assumed, it could be shown by Southern blot analysis that there is not a complete double strand, because the biotinylated primer which hybridizes to the phosphorylated strand did not bind (Figure 2B, lane 3-7). However, the dsDNA without digestion exhibited biotinylated primer binding (Figure 2B, lane 1). The incubation of polyA ssDNA library (pA_SB) with phosphate labeled primer (Figure2A, lane 8) demonstrated that the binding of phosphate primer to the library leads to the similar migration behaviour as the dsDNA in non-denaturing PAGE and thereby appears at the same height as the dsDNA. In the samples after 30 to 90 minutes digestion, the Southern blot analysis demonstrated a visible smear under the desired strand (polyA_strand) (Figure 2B, lane 3-5) which indicates a not fully completed digestion of the phosphorylated DNA strand. The biotinylated primer for detection of phosphorylated DNA strand binds at the end of this strand. If it is completely digested, the primer cannot bind to the DNA anymore because of the missing hybridization sequence. The smear disappeared in samples with 2 h or 4 h digestion (Figure 2B, lane 6 and 7) which demonstrates that the digestion is completed after 2 h.

**Figure 3:**
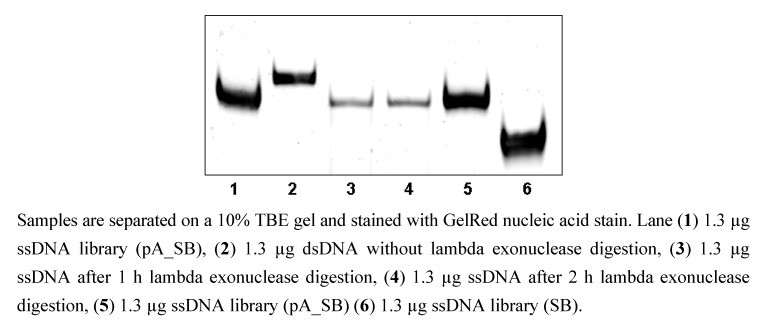
Digestion of the phosphorylated strand of dsDNA with 25 U lambda exonuclease after PCR with 1 ng template per reaction.

To eliminate the weak band which presents a polyA strand with bound phosphate primer (Figure 2A, lane 3-7), the PCR was performed with reduced template amount. The use of 1 ng DNA template (ssDNA library (pA_SB)) per 100 µL PCR reaction led to the disappearing of this band (Figure 3, lane 3 and 4). The ssDNA amount after five independent strand separation experiments with 25 U lambda exonuclease per 6.6 µg dsDNA and 2 h digestion at 37 °C was determined. The average yield was 62.3 ± 8.3% from the maximum possible ssDNA amount.

After strand separation with streptavidin-coated beads, the biotinylated DNA strand in the aptamer pool can be directly detected after blotting via alkaline phosphatase-conjugated anti-biotin antibody. However, after lambda exonuclease digestion, due to the absence of biotin in the phosphorylated ssDNA strand, the presence of complementary strand can only be detected using the hybridization of a biotinylated primer and subsequent detection by alkaline phosphatase-conjugated anti-biotin antibody. Thereby, the sensitivity of both detection methods can differ. However, the non-radioactive Southern blot method is one of the most sensitive and specific method for detection of complementary strand after lambda exonuclease digestion. Using this method, we could demonstrate a digestion time dependent decrease of phosphorylated strand. After 2 h digestion, the non-desired complementary strand was no more detectable. Moreover, the lambda exonuclease digestion provided high yield of ssDNA.

For the following selection cycles, it is important that the ssDNA pool does not contain any lambda exonuclease. Therefore, we performed after lambda exonuclease digestion phenol/chloroform extraction and subsequent ethanol precipitation to eliminate the lambda exonuclease from the aptamer pool. Thereby, the presence of an additional SELEX target molecule such as lambda exonuclease during selection is prevented. However, although this step eliminates lambda exonuclease, it is also associated with a loss of approximately 30% ssDNA. Nevertheless, we could generate an average yield of 62.3 ± 8.3% ssDNA from the maximum possible ssDNA amount. This method yields the highest ssDNA amount than all other ssDNA production methods which have been mentioned in this work. However, it should be noted that the yield and purity of ssDNA is influenced by the quality of PCR product. Therefore, PCR conditions (such as template amount, PCR protocol, primer concentration and number of cycles) should be optimized to obtain only one defined length of dsDNA. Design of the oligonucleotide library such as the size of the randomized region and primer-binding sequences flanking the randomized region also plays an important role for generation of only a single dsDNA product. The primer region should be long enough (20–24 nucleotides) to ensure stable and specific hybridization of primers at the required temperature. The two primers for the amplification should not contain regions of internal complementary sequences to avoid hairpin structures. In addition, regions of sequence overlap with another primer should be avoided to circumvent primer-dimer formation. A non-observance of these points may lead to the generation of longer or smaller size PCR products, thereby a complete lambda exonuclease digestion of all phosphorylated strands cannot be ensured. The enzyme has greatly reduced activity on ssDNA and non-phosphorylated dsDNA and no activity against nicked or gapped DNA [24,25]. If using heterogeneous template sequences such as the ssDNA library in the PCR for the selection of aptamers, the different GC contents in the sequences can cause non-uniform amplification during PCR due to the stable stem-loop secondary structure formation of GC-rich regions [26]. These structures promote polymerase jumping during PCR amplification which yields PCR products of smaller sizes missing the stem-loop region of the template [27]. These undesired products can be eliminated by performing PCR with 5% DMSO [28] and 1 mM betaine [29]. Thereby, the yield of dsDNA is reduced but the quality is enhanced.

Another important role for complete lambda exonuclease digestion of phosphorylated DNA strand plays the manufacturing of phosphorylated primer. Phosphate groups are temporarily protected by blocking groups until the oligonucleotide synthesis is completed. After termination of synthesis, these groups must be completely removed from the phosphate groups to enable entire lambda exonuclease digestion of phoshorylated DNA strands.

## Experimental

### ssDNA library and primers

The ssDNA libraries (pA_SB: 5´-polyA(20)-[spacer-C18]-GCCTGTTGTGAGCCTCCTAAC-N49-CATGCTTATTCTTGTCTCCC-3´) and (SB: 5´-phosphate-GCCTGTTGTGAGCCTCCTAAC-N49-CATGCTTATTCTTGTCTCCC-3´) comprised a randomized sequence region of 49 nucleotides (N) in the center flanked by fixed primer hybridization regions. PCR was performed with a forward primer that contains a 18-carbon ethylene glycol spacer as terminator and an extension of 20 adenine nucleotides (polyA(20)) at its 5´-end to produce strands with different lengths (P1_pA: 5´-polyA(20)-[spacer-C18]-GCCTGTTGTGAGCCTCCTAAC-3´). The reverse primer was 5´-phosphorylated to perform lambda exonuclease digestion (P2_phosphate: 5´-phosphate-GGGAGACAAGAATAAGC- ATG-3´). In contrast, the reverse primer for DNA strand separation with streptavidin-coated magnetic beads was 5´-biotin labeled to enable the binding of dsDNA to streptavidin-coated magnetic beads (P2_biotin: 5´-biotin-GGGAGACAAGAATAAGCATG-3´). For detection of phosphorylated strand by Southern blot 5´-biotin labeled forward primer was used (P1_biotin: 5´-biotin-GCCTGTTGTGAG- CCTCCTAAC-3´). All primers and the start library were HPLC-purified and purchased from Ella Biotech GmbH (Martinsried, Germany).

### PCR

#### PCR for generation of phosphate labeled dsDNA

PCR was performed with 100 or 1 ng template (ssDNA library (pA_SB)) per PCR reaction. All PCR reactions were carried out in a volume of 100 µL with forward primer (P1_pA) and reverse primer (P2_phosphate) as follows. An initial denaturing step of 95 °C for 3 minutes was used to completely denature the template DNA. This was followed by 25 rounds standard PCR protocol, with a denaturing step at 95 °C for 45 seconds, an annealing step at 58 °C for 45 seconds and an elongation step at 72 °C for 1 minute. A final extension step was performed at 72 °C for 5 minutes. The PCR products were purified by QIAquick PCR purification kit (Qiagen, Hilden, Germany).

#### PCR for generation of biotin labeled dsDNA

PCR was performed with 1 ng template (ssDNA library (pA_SB)) per PCR reaction. All PCR reactions were carried out in a volume of 100 µL with forward primer (P1_pA) and reverse primer (P2_biotin) as described above. The PCR products were purified by QIAquick PCR purification kit (Qiagen, Hilden, Germany).

### ssDNA generation

#### Strand separation with streptavidin-coated magnetic beads and alkaline denaturation

After PCR with biotinylated reverse primer, the strand separation was performed with streptavidin-coated magnetic beads (M-280 Dynabeads; Invitrogen, Karlsruhe, Germany). The experiments were performed with this brand since these beads are most commonly used by many research groups for DNA strand separation during SELEX experiments. The beads were prepared according to manufacturers´ instructions. From the purified PCR product, 50 µg double stranded DNA was incubated for 25 minutes on a rotator with 5 mg pre-washed beads. After three washes with binding and washing buffer (10 mM Tris-HCl pH 7.5, 1 mM EDTA, 2 M NaCl), an alkaline denaturation was performed with 100 µL freshly prepared 0.15 M NaOH for 2 minutes. The eluate was neutralized by titration with 0.15 M HCl and filtrated through a Spin-X centrifugation filter (0.22 µm, Corning, USA) to remove all beads which remain in the eluate since the last magnetization step is not strong enough. After centrifugation at 13,000 rpm for 1 minute, the ssDNA was concentrated by ethanol precipitation. The dsDNA before strand separation and the generated ssDNA product were separated by denaturing PAGE (10% TBE-Urea Gel). All electrophoresis reagents were obtained from Biorad (Munich, Germany).

### Lambda exonuclease digestion

After PCR with phosphate labeled reverse primer, 6.6 µg purified dsDNA was incubated with 25 U lambda exonuclease (New England Biolabs, Frankfurt am Main, Germany) in a total reaction volume of 50 µL in 1× lambda exonuclease reaction buffer at 37 °C. The reaction was terminated after different digestion times (0, 30, 60, 90, 120, and 240 minutes) by 10 minutes incubation at 75 °C. From each reaction mixture 10 µL samples were taken out and loaded on 10% non-denaturing polyacrylamide gels and separated. The gels were stained with GelRed nucleic acid stain (Biotium, Hayward, USA). All electrophoresis reagents were obtained from Biorad (Munich, Germany).

### Detection of phosphorylated DNA strand after lambda exonuclease digestion

#### Non-radioactive Southern Blotting

The DNA was transferred from 10% non-denaturing TBE gels onto positively charged nylon membranes (Biobond plus membrane, Sigma-Aldrich, Munich, Germany) using electroblot method. The transferred DNA on nylon membranes was denatured with 0.4 M NaOH for 20 minutes. Thereafter, the membrane was baked at 80 °C for 30 minutes to permanently affix the DNA to the membrane. The membrane was incubated for 2 hours in prehybridization solution (50% formamide, 6× SSC-buffer, 5× Denhardt’s reagent, 0.5% SDS and 50 µg/mL salmon sperm DNA) at 42 °C. Then, the membrane was incubated over night at 42 °C with hybridization solution (consists of prehybridization solution with 2 nmol biotinylated forward primer (P1_biotin)). This primer binds to the 3´-end of phosphorylated DNA strand, if it is present. Next day, the membrane is washed 2 times for 5 minutes at room temperature with wash solution 1 (2× SSC/0.1% SDS) and 3 times for 10 minutes at 42 °C with wash solution 2 (0.2× SSC/0.1% SDS). The blots were blocked in 3% nonfat dry milk (Sigma-Aldrich, Munich, Germany) and incubated for 2 hours at room temperature with alkaline phosphatase conjugated goat polyclonal anti-biotin antibody (1:1,000 dilution) (abcam, Cambridge, UK). Subsequently, the membranes were incubated with the BCIP/NBT (5-bromo-4-chloro-3-indolyl phosphate/nitro blue tetrazolium) solution (Sigma-Aldrich, Munich, Germany) for colorimetric detection of alkaline phosphatase-conjugated antibodies.

### Detection of biotinylated DNA strand after strand separation with streptavidin-coated magnetic beads and alkaline denaturation

#### Modified Western blot

The separated DNA was transferred from 10% TBE-Urea gel onto positively charged nylon membranes (Biobond plus membrane, Sigma-Aldrich, Munich, Germany) using electroblot method. The blots were blocked in 3% nonfat dry milk (Sigma-Aldrich, Munich, Germany) and incubated for 2 hours at room temperature with alkaline phosphatase conjugated goat polyclonal anti-biotin antibody (1:1,000 dilution) (abcam, Cambridge, UK). Subsequently, the membranes were incubated with the BCIP/NBT solution (Sigma-Aldrich, Munich, Germany) for colorimetric detection of alkaline phosphatase-conjugated antibodies.

## Conclusions

Preparation of ssDNA is an essential and important step in the combinatorial chemistry technique SELEX for *in vitro* selection of ssDNA aptamers and numerous other molecular biology procedures. Our studies demonstrated that the ssDNA generation by lambda exonuclease digestion is superior to other techniques. Lambda exonuclease selectively digests the phosphorylated undesired strand of dsDNA and produces ssDNA in high yield and purity. We strongly believe that the application of this ssDNA generation method during *in vitro* selection of ssDNA aptamers, particularly during cell-SELEX, will greatly increase the success of the SELEX technology.
